# F‐box protein FBXO16 functions as a tumor suppressor by attenuating nuclear β‐catenin function

**DOI:** 10.1002/path.5252

**Published:** 2019-03-08

**Authors:** Debasish Paul, Sehbanul Islam, Rajesh Kumar Manne, US Dinesh, Sunil K Malonia, Biswanath Maity, Ramanamurthy Boppana, Srikanth Rapole, Praveen Kumar Shetty, Manas Kumar Santra

**Affiliations:** ^1^ Cancer Biology division, National Centre for Cell Science Pune India; ^2^ Department of Biotechnology Savitribai Phule Pune University Pune India; ^3^ Department of Biochemistry/Central Research Laboratory SDM College of Medical Sciences and Hospital Dharwad India; ^4^ Department of Molecular, Cell and Cancer Biology University of Massachusetts Medical School Worcester MA USA; ^5^ Centre of Biomedical Research Lucknow India

**Keywords:** FBXO16, tumor suppressor, β‐catenin, Wnt signaling, proteasomal degradation

## Abstract

Aberrant activation of β‐catenin has been implicated in a variety of human diseases, including cancer. In spite of significant progress, the regulation of active Wnt/β‐catenin‐signaling pathways is still poorly understood. In this study, we show that F‐box protein 16 (FBXO16) is a putative tumor suppressor. It is a component of the SCF (SKP1‐Cullin1‐F‐box protein) complex, which targets the nuclear β‐catenin protein to facilitate proteasomal degradation through the 26S proteasome. FBXO16 interacts physically with the C‐terminal domain of β‐catenin and promotes its lysine 48‐linked polyubiquitination. In addition, it inhibits epithelial‐to‐mesenchymal transition (EMT) by attenuating the level of β‐catenin. Therefore, depletion of FBXO16 leads to increased levels of β‐catenin, which then promotes cell invasion, tumor growth, and EMT of cancer cells. Furthermore, FBXO16 and β‐catenin share an inverse correlation of cellular expression in clinical breast cancer patient samples. In summary, we propose that FBXO16 functions as a putative tumor suppressor by forming an SCF^FBXO16^ complex that targets nuclear β‐catenin in a unique manner for ubiquitination and subsequent proteasomal degradation to prevent malignancy. This work suggests a novel therapeutic strategy against human cancers related to aberrant β‐catenin activation. © 2019 The Authors. *The Journal of Pathology* published by John Wiley & Sons Ltd on behalf of Pathological Society of Great Britain and Ireland.

## Introduction

Cancer is one of the leading causes of death, and breast cancer contributes to a majority of the mortality rate among women 1, 2. Among different subtypes of breast cancer, triple‐negative breast cancer (TNBC) is associated with poor prognosis 3.

Previous reports suggested the activation of Wnt signaling in 50% of breast cancer cases, with a relatively lower frequency of somatic mutations in Wnt signaling components 4, 5, 6. Furthermore, Wnt ligands and receptors involved in canonical Wnt signaling are often highly expressed in breast cancer, whereas secreted antagonists are attenuated 7, 8, 9, 10. Aberrant activation of Wnt signaling results in the nuclear accumulation of β‐catenin, which is associated with invasiveness of breast cancer with poor outcomes 11, 12, 13. Nuclear β‐catenin functions as a transcriptional coactivator of the TCF/LEF complex, promoting the transcriptional activation of many oncogenes associated with increased growth, invasion, and cellular transformation 14, 15, 16. β‐Catenin is majorly regulated by the ubiquitin proteasome system (UPS), which directs the proteolysis through polyubiquitylation by E3 ubiquitin ligases (E3Ls). Several reports suggest that multiple E3 ubiquitin ligases are involved in the proteasomal degradation of β‐catenin 17, 18, 19.

It is well known that the proteasomal degradation of cellular proteins is mostly controlled by SCF (SKP1‐Cullin1‐F‐box protein) E3 ubiquitin ligases. F‐box proteins are an integral component of the SCF complex and function as substrate receptors. Human genome encodes 69F‐box proteins; however, the function of a majority of the F‐box proteins remains elusive 20. Notably, deregulation of F‐box protein‐mediated proteasomal degradation is often associated with malignancy 21.

Here, we decipher the function of FBXO16, an F‐box protein, in cancer. In an attempt to identify interacting proteins of FBXO16, we adopted a mass spectrometry approach. We identified β‐catenin as a vital cellular substrate of FBXO16. Our results show that FBXO16 is a putative tumor suppressor that suppresses the growth, migration, invasion of cancer cells, and EMT by directing the proteasomal degradation of nuclear β‐catenin. Interestingly, the expression of FBXO16 and β‐catenin is conversely correlated in breast cancer patient samples, with the attenuation of FBXO16 expression with cancer progression. Altogether, these findings might contribute to developing new therapeutic targets for limiting nuclear β‐catenin‐mediated malignancy.

## Materials and methods

### Cell culture

The human cell lines MCF10A, MCF7, MDA‐MB‐231; T47D and HCT‐116; and HEK293 and HEK293T were kind gifts from Prof. Michael Green (UMass Medical School, MA, USA). MCF7, HEK‐293, and HEK‐293 T cells were cultured at 37 °C in DMEM (Gibco, Waltham, MA, USA). MDA‐MB‐231, T47D, and HCT‐116 were maintained in RPMI‐1640. MCF10A cells were grown in DMEM‐F12 (Gibco) media. All tissue culture media were supplemented with 10% FBS/horse serum and 2 mm l‐glutamine and were cultured in 5% CO_2_ at 37 °C in a humidified atmosphere.

### Plasmid transfection

GFP‐WT‐β‐catenin, GFP‐Δ*N*‐β‐catenin, GFP‐ΔC‐β‐catenin, GFP‐ΔNC‐β‐catenin, and GFP‐T41A‐β‐catenin were kind gifts from Dr. Jomon Joseph, Pune, Maharashtra, India. psPAX2 and pMD2.G were obtained from Didier Trono (Addgene, Watertown, MA, USA 12260, 12259). Human cDNA encoding FBXO16 was procured from Origene (Rockville, MA, USA). The F‐box deletion mutant was generated using the primers shown in supplementary material, Table S1. Plasmids were transfected using polyethylenimine (PEI) as described previously 22.

### Cell lysate preparation and immunoblotting

Cells were harvested, washed with PBS, and lysed with buffer on ice for 30 min 23. Cell lysates were centrifuged at 16 000 × *g*, and supernatants were collected. Cellular fractionation was performed as described previously 24. Protein concentration was measured using Bradford's method 25. Protein samples were immunoblotted with the indicated antibodies: those for α‐tubulin (T5168) and phospho β‐catenin (T41) (C‐8616) were procured from Sigma (St. Louis, MO, USA) and for ubiquitin (sc‐8017), GFP (sc‐9996), SKP1 (sc‐5281), Cullin 1 (sc‐17775), PARP1 (sc‐7150), Cyclin D1 (sc‐8396), β‐Trcp (sc‐390629), Twist (sc‐81417), and Snail (sc‐271977) from Santa Cruz Biotechnology, Dallas, Texas, USA and those for β‐catenin (610154) and e‐cadherin (610181) were from BD Biosciences (San Diego, CA, USA). Antibodies to c‐Myc (9402), phospho GSK3β (8566), phospho PKCδ (9376), and K48‐linked ubiquitin antibodies (8081) were from Cell Signaling Technology (Danvers, MA, USA); to DDK (clone OTI4C5) from Origene; and FBXO16 (PA5‐31419) was from Thermo Fisher Scientific (Waltham, MA, USA).

### Coimmunoprecipitation

Immunoprecipitation was performed using 600 μg proteins and 2 μg of antibody in IP lysis buffer as described previously 23. Immunoprecipitates were immunoblotted as described above, and 3% of protein extracts was used as input.

### Identification of FBXO16 interactomes

MCF7 cells expressing either vector or DDK‐FBXO16 were grown in 5 μm MG132 for 6 h before harvesting. Cells were lysed, and protein extracts were immunoprecipitated with anti‐DDK antibody 23. The immunoprecipitates were eluted from the beads using elution buffer (8 M urea and 0.5 M Tris–pH 7.4) and were processed for mass spectrometry. An LC–MS (liquid chromatography–mass spectrometry)/MS analysis of peptide mixture was performed using Orbitrap fusion mass spectrometer (Thermo Fisher Scientific).

### Generation of stable knockdown cells

Lentiviral shRNAs (For FBXO16, shRNA 1: V2LHS_118578 and shRNA 2: V2LHS_118583) were a kind gift from Prof. Michael Green (UMass Medical School, Worcester, MA, USA). Stable knockdown cells were generated by lentivirus transduction as described previously 24.

### Colony formation assay

A total of 5000 cells (transduced/treated) were seeded in 35 mm‐diameter dishes and allowed to grow for 12–15 days to form colonies. Cells were fixed with 3.7% formaldehyde and stained with 0.05% crystal violet. Plates were photographed, and representative images are shown in Figure 6A and see supplementary material, Figure S6A.

### Soft agar assays

Soft agar assays were performed as described previously 26. In brief, 35 mm‐diameter dishes were filled with 0.6% base agar (Invitrogen, Carlsbad, CA, USA; Cat‐75 510‐019) and 2× RPMI 1640 (MDA‐MB‐231 cells)/2× DMEM (MCF7 cells) with 20% FBS and allowed to solidify. Thereafter, 5000 cells suspended in 0.3% of agar containing 20% FBS were placed on the top of the base agar. Twenty days later, cells were observed under a microscope and photographed.

### Migration and invasion assays

Scratch wound‐healing assay was performed as described previously 27. In brief, cells were seeded and were allowed to grow as a confluent monolayer. A scratch‐mediated wound was made in the presence of 5 ng/ml of actinomycin D, and the open space was tracked continuously using a phase‐contrast microscope (Olympus IX71, Shinjuku, Tokyo, Japan).

Invasion assays were performed as described previously 28. In brief, cells were serum‐starved for 24 h, and 50 000 cells were then suspended in 200 μl of media containing 0.5% FBS in the upper chamber. Media containing 10% FBS was added to the lower chamber. After 16 h of culturing, invaded cells were fixed with 3.7% formaldehyde, stained with 0.5% crystal violet, different fields photographed, and the number of invading cells was expressed as the average number of cells per microscopic field.

### RT‐qPCR

RT‐qPCR was performed using SYBRmix as described previously 24. *GAPDH* was used to normalize the data. The primers used are listed in supplementary material, Table S2.

### Ubiquitination assays

*In vitro and in vivo* ubiquitination assays were performed as described previously 23. The immunoprecipitates (*in vivo*) and ubiquitylation reaction mixture (*in vitro*) were resolved by SDS‐PAGE and blots probed with an ubiquitin‐specific antibody (we used: His‐tag, K48‐linked, and those mentioned in respective figure captions).

### Immunohistochemistry

Tissue samples were obtained from SDM College of Medical Sciences following established protocols and with Institutional Ethical Board approval. Tissue samples were stained with H&E, and the histological type and grade of tumors were determined. β‐Catenin and FBXO16 levels in breast cancer patients, including malignant tissue and adjacent nonmalignant epithelium, were detected using immunohistochemical staining and were quantified as described previously 24.

### TOP/FOP assay

Cells were seeded in a 24‐well plate. Cells were transduced/transfected with the indicated plasmids for 60 or 36 h, respectively. Cells were then treated with either EGF or Wnt3a‐conditioned medium for 12 h prior to harvesting. Luciferase activity was measured according to the manufacturer's protocol (Thermo Fisher Scientific, Cat. no‐16176), and Renilla luciferase was used for normalization.

### Chromatin immunoprecipitation assay

Chromatin immunoprecipitation (ChIP) assay was performed as described previously 29. In brief, cells expressing vector or DDK‐FBXO16 were cross‐linked with 3.7% formaldehyde for 15 min, followed by quenching with 125 mm glycine. Cells were then washed with PBS and lysed. The cell lysates were sonicated and then incubated overnight with anti‐β‐catenin antibody at 4 °C. Preblocked protein A agarose beads were incubated with lysate–antibody complex for 2 h, followed by reversal of cross‐linking using proteinase K. DNA was extracted using a phenol–chloroform method. qPCR was performed using indicated primers listed in supplementary material, Table S3.

### Xenograft assays

MCF7 cells (5 × 10^6^ cells/mice) stably expressing NS and *FBXO16* shRNA were injected subcutaneously in the right flank of 5‐week‐old NOD‐SCID mice (*n* = 5). The length (*L*) and width (*W*) of tumors were measured with a digital Vernier caliper, and tumor volume was calculated using the formula: *V* = (*L* × *W* ^2^)/2, with W being smaller than *L*. All animal experiments were approved by the Institutional Animal Ethics Committee of the National Centre for Cell Science.

### Statistical analysis

Each experiment was repeated at least thrice. Values are shown as mean ± SD unless otherwise mentioned.

## Results

### FBXO16 interacts with β‐catenin

The cellular function of FBXO16 is poorly understood. To discover its cellular function and physiological substrates, we performed coimmunoprecipitation followed by mass spectrometry to identify its interacting partners. Results demonstrated that FBXO16 interacts with 117 cellular proteins, and β‐catenin was found to be the most represented candidate, with coverage of six unique peptides (see supplementary material, Figure S1A and Table S4).

**Figure 1:**
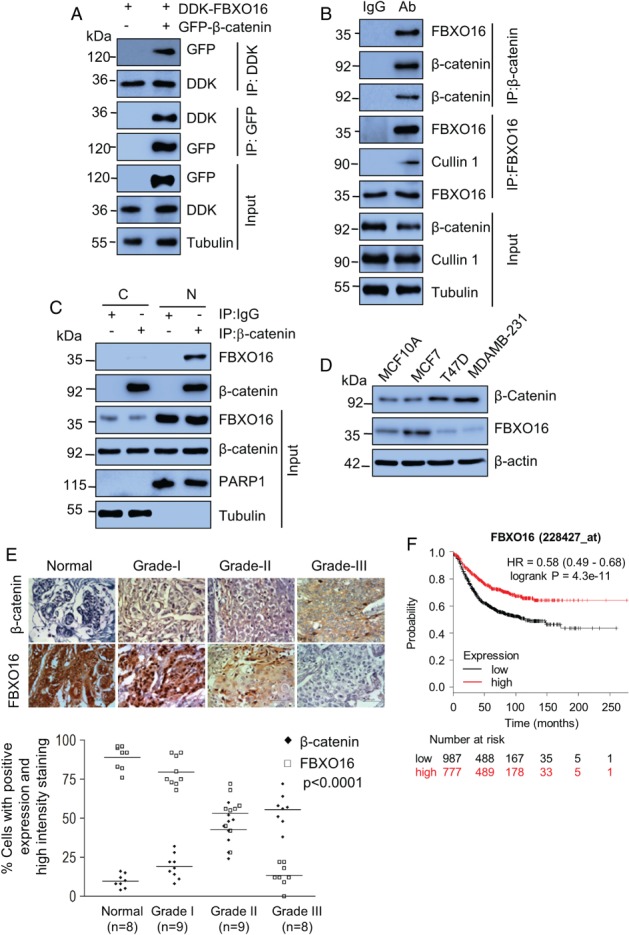
FBXO16 interacts with β‐catenin. (A) MCF7 cells coexpressing DDK‐FBXO16, either with vector control or GFP‐β‐catenin for 40 h, were then incubated with 5 μm MG132 for 6 h. Whole‐cell lysates were immunoprecipitated with the indicated antibodies. Immunoprecipitates and input lysates were separated on SDS‐PAGE and immunoblotted for the indicated proteins. Tubulin was used as a loading control throughout our study (*n* = 3). (B) Whole‐cell lysates of MCF7 cells, pretreated with 5 μm MG132 for 6 h before harvesting, were immunoprecipitated with the indicated antibodies to pull‐down endogenous proteins. IgG immunoprecipitates were used as a control. Immunoprecipitates and input lysates were separated on SDS‐PAGE and immunoblotted for the indicated proteins (*n* = 2). (C) Nuclear and cytoplasmic fractions were immunoprecipitated with either anti‐β‐catenin antibody or IgG. Immunoprecipitates and input lysates were separated on SDS‐PAGE and immunoblotted for the indicated proteins (*n* = 2). Cells were grown in the presence of 5 μm MG132 for 6 h before harvesting. (D) Whole‐cell protein extracts of MCF10A, MCF7, T47D, and MDA‐MB‐231 cells were immunoblotted for β‐catenin, FBXO16, and tubulin (*n* = 3). β‐actin was used as a loading control. (E) (Top) Expression levels of β‐catenin (top row) and FBXO16 (bottom) in samples of normal and different grades of breast cancer. Tissues were immunostained as described in the ‘Materials and methods’ section. Lower panel: the statistical analysis of the average score of β‐catenin and FBXO16 staining between cancer tissues and corresponding nontumor tissues, *p* < 0.0001 (one‐way ANOVA). Staining intensity of these proteins in neoplastic cells was graded on a scale of 0 (no staining) to 3+ (strong staining). The protein expression was scored based on the percentage of positive cells: 0 = 0% of stained positive cells; 1 = weakly stained tissue or 1–25% of positive cells; 2 = moderate stained tissue or 26–50% of positive stained cells; and 3 = strongly stained tissue or more than 50% of stained cells. (F) Kaplan–Meier plot depicting overall survival of breast cancer patient cohorts with different levels of FBXO16 expression. Red trace represents comparative higher expressions of FBXO16.

Next, we performed coimmunoprecipitation experiments to validate the mass spectrometry finding. As shown in Figure 1A, GFP‐β‐catenin could be detected in the DDK‐FBXO16 immunoprecipitates, and conversely, DDK‐FBXO16 could be detected in the GFP‐β‐catenin immunoprecipitates. Similar results were obtained at the endogenous level (Figure 1B). Notably, the presence of cullin‐1 in the FBXO16 immunoprecipitates indicated that FBXO16 is part of an SCF complex (Figure 1B). Likewise, we also found FBXO16 and β‐catenin interaction in an unrelated cell line HEK‐293, which was increased following the addition of proteasome inhibitor MG132 (see supplementary material, Figure S1B). We also confirmed the interaction *in vitro* using purified His‐FBXO16 and GST‐β‐catenin (see supplementary material, Figure S1C). Subcellular localization results demonstrated that FBXO16 predominantly localizes (Figure 1C and see supplementary material, Figure S1D) and interacts with β‐catenin in the nucleus (Figure 1C). Results taken together confirmed that β‐catenin is an interacting partner of FBXO16.

β‐Catenin is known to be involved in progression and metastasis of breast cancer, and we were intrigued to draw up a correlation between β‐catenin and FBXO16 in the context of breast cancer. Accordingly, using different breast cell lines, we discovered an inverse relationship between β‐catenin and FBXO16 levels (Figure 1D). For a clinical perspective, we then checked the correlation of FBXO16 and β‐catenin expression in the breast cancer patient samples. Immunohistochemical analysis demonstrated a significant converse correlation of FBXO16 and β‐catenin expression with increased levels of β‐catenin and concomitant attenuation of FBXO16 in higher grades of breast cancer patient samples (Figure 1E). Interestingly, a similar relationship was also observed irrespective of hormonal status of breast cancer patients (see supplementary material, Figure S1E). The attenuation of FBXO16 was further supported by the Oncomine database (see supplementary material, Figure S1F). Furthermore, the TCGA database demonstrated a close association of higher FBXO16 expression with disease‐free survival (Figure 1F and see supplementary material, Figure S1G). Collectively, our findings suggested that FBXO16 might function as a putative tumor suppressor by limiting nuclear β‐catenin activity.

### FBXO16 promotes ubiquitylation and proteasomal degradation of β‐catenin

**Figure 2:**
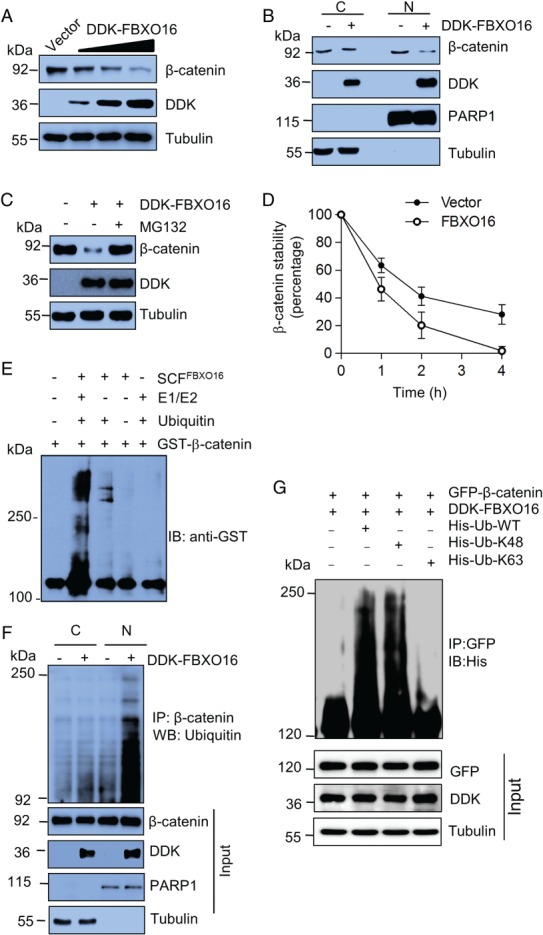
FBXO16 promotes proteasomal degradation of β‐catenin. (A) Ectopically expressed DDK‐FBXO16 decreased the levels of β‐catenin in a dose‐dependent manner. Whole‐cell lysates of MDA‐MB‐231 cells expressing either vector control or increasing doses of DDK‐FBXO16 were immunoblotted with the indicated antibodies (*n* = 3). MDA‐MB‐231 cells were transfected using Lipofectamine 3000 according to the manufacturer's protocol throughout our study. (B) Fractionated cell lysates of MDA‐MB‐231 cells expressing either vector control or DDK‐FBXO16 were immunoblotted using the indicated antibodies. PARP1 and tubulin were used as nuclear and cytoplasmic loading controls, respectively (*n* = 3). (C) MDA‐MB‐231 cells were transfected with either vector control or DDK‐FBXO16 for 42 h. FBXO16‐transfected cells were then incubated with or without 5 μm MG132 for 6 h. Whole‐cell lysates were immunoblotted for the indicated proteins (*n* = 4). (D) Levels of β‐catenin (in cycloheximide assay, see supplementary material, Figure S2C) were quantified and normalized to the loading control. Expression levels of β‐catenin were then normalized to 100% at time 0 h. (E) *In vitro* ubiquitylation assay showed that FBXO16 promotes ubiquitylation of β‐catenin. Purified FBXO16, GST‐ β‐catenin, ubiquitin, and E1, E2 enzymes were incubated together and immunoblotted for anti‐GST antibody. (F) Cytoplasmic (C) and nuclear (N) protein extracts of MDA‐MB‐231 cells expressing either vector control or DDK‐FBXO16 were immunoprecipitated with β‐catenin. Immunoprecipitates and input protein extracts were immunoblotted for indicated proteins. Cells were treated with MG132 (10 μm for 4 h) prior to lysis. PARP1 and tubulin were used as nuclear and cytoplasmic loading controls, respectively (*n* = 2). (G) FBXO16 promotes K48‐linked polyubiquitylation of β‐catenin. Whole‐cell lysates of MCF7 cells coexpressing GFP‐β‐catenin and DD‐FBXO16 along with HIS‐K63/HIS‐K48 Ub for 48 h were immunoprecipitated with anti‐GFP antibody, followed by immunoblotting for His. Whole‐cell lysates were immunoblotted for the indicated proteins. The experiment was repeated three times.

Being an F‐box family member, we presumed that FBXO16 might regulate the stability of β‐catenin at the posttranslational level, and to test that, we performed a series of experiments. First, the dose‐dependent ectopic expression of FBXO16 in MDA‐MB‐231 cells decreased β‐catenin levels (Figure 2A). Corroborating with our finding of FBXO16's nuclear localization, we found a significant attenuation of nuclear β‐catenin following ectopic expression of FBXO16 (Figure 2B, supplementary material, Figure S2A). However, RT‐qPCR results suggested that *CTNNB1* mRNA levels were unaffected by ectopic expression of FBXO16 (see supplementary material, Figure S2B). Furthermore, FBXO16‐mediated reduction of β‐catenin was significantly blocked in the presence of MG132, indicating that β‐catenin stability is indeed regulated at the posttranslational level by FBXO16 through the 26S proteasome (Figure 2C). In addition, a cycloheximide chase assay demonstrated that the turnover of β‐catenin was increased on FBXO16 expression (Figure 2D, supplementary material, Figure S2C).

To demonstrate FBXO16's ubiquitin‐conjugating action irrefutably, polyubiquitylated levels of β‐catenin were examined. An *in vitro* polyubiquitination assay demonstrated that FBXO16 promotes polyubiquitylation of β‐catenin (Figure 2E). Furthermore, Figure 2F shows the presence of a notable high mass ladder of ubiquitylated β‐catenin exclusively in the nuclear fraction, suggesting that FBXO16 facilitates proteasomal degradation of nuclear β‐catenin. In addition, Figure 2G and supplementary material, Figure S2D, show that polyubiquitin chains on β‐catenin are lysine 48 (K48)‐linked, which substantiates the widely asserted principle that K48‐based polyubiquitylation on substrate leads to 26S proteasome‐mediated degradation.

### FBXO16 directs β‐catenin degradation through an SCF complex

**Figure 3:**
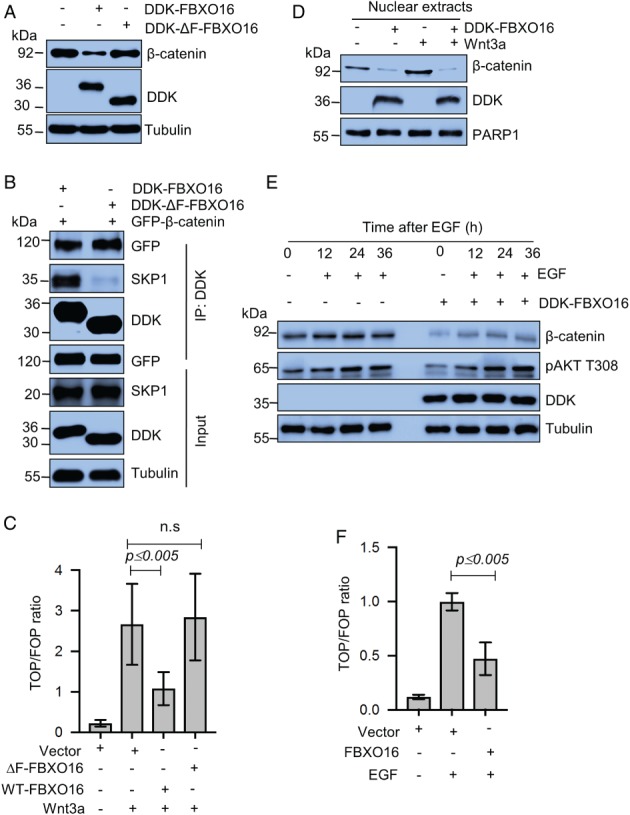
SCF^FBXO16^‐mediated proteasomal degradation of β‐catenin is independent of Wnt/EGF signaling. (A) Whole‐cell protein extracts of MDA‐MB‐231 cells expressing either vector control or DDK‐FBXO16 or DDK‐ΔF‐FBXO16 for 48 h were immunoblotted for the indicated proteins (*n* = 3). (B) MDA‐MB‐231 cells were coexpressing GFP‐β‐catenin either with DDK‐FBXO16 or DDK‐ΔF‐FBXO16 for 36 h. Transfected cells were then grown in the presence of 5 μm MG132 for 6 h. Whole‐cell lysates were immunoprecipitated with anti‐DDK antibody. Immunoprecipitates and input protein extracts were resolved in SDS‐PAGE and immunoblotted for the indicated proteins (*n* = 3). (C) MDA‐MB‐231 cells were transfected with the indicated plasmids, including TOP/FOP and pRL‐TK. Transfection efficiency was normalized using the pRL‐TK reporter. Luciferase activity was measured at 48 h posttransfection. After 36 h of transfection, cells were treated with Wnt3a for 12 h. Luciferase activity is shown as a ratio of TOP and FOP. ‘ns’ means statistically not significant (*n* = 3). (D) Nuclear extracts of MDA‐MB‐231 cells expressing either vector control or DDK‐FBXO16 for 48 h were immunoblotted for the indicated proteins. At 36 h posttransfection, cells were treated with Wnt3a for 12 h (*n* = 4). (E) Whole‐cell lysates of MDA‐MB‐231 cells expressing either vector control or DDK‐FBXO16 in the absence and presence of EGF for the indicated time periods were immunoblotted for the indicated proteins. (F) MDA‐MB‐231 cells were transfected with the indicated plasmids, including TOP/FOP and pRL‐TK. At 36 h posttransfection, cells were treated with EGF for 12 h, (*n* = 3). Luciferase activity was measured at 48 h posttransfection, and transfection efficiency was normalized using the pRL‐TK reporter. Luciferase activity is shown as a ratio of TOP and FOP (*n* = 3).

Typically, F‐box proteins associate with SKP1 to form an SCF complex through their F‐box motif and bring their substrates to the complex for ubiquitylation. To examine whether FBXO16 complies with this, wild‐type and F‐box‐deleted FBXO16 (ΔF‐FBXO16) were overexpressed in MDA‐MB‐231 cells. Results demonstrated that ΔF‐FBXO16 is incompetent to reduce the expression of β‐catenin (Figure 3A). Figure 3B explicitly illustrates that ΔF‐FBXO16 did interact with its substrate, but not with SKP1, to form the fully functional SCF complex. This observation was further supported by polyubiquitylation assay (see supplementary material, Figure S3A). Interestingly, we identified the presence of a potential nuclear localization signal (NLS) at the C‐terminus of FBXO16 (247–273 aa). Immunoblotting results demonstrated that an NLS‐deleted mutant (DDK‐ΔNLS‐FBXO16) was predominantly localized in the cytoplasm (see supplementary material, Figure S1D) and could not degrade nuclear β‐catenin (see supplementary material, Figure S3B), suggesting that the NLS of FBXO16 is important for nuclear β‐catenin degradation.

### FBXO16‐mediated β‐catenin degradation is independent of its activating signals

Wnt ligands are the canonical activators of β‐catenin 30, 31 and thus encouraged us to investigate whether Wnt3a stimulation averts FBXO16‐facilitated degradation of β‐catenin. We performed T‐cell factor/β‐catenin transcription assays (TOP/FOP assay) to assess the transactivation of β‐catenin, which demonstrated that FBXO16‐mediated β‐catenin's degradation occurs even under Wnt3a induction (Figure 3C and supplementary material, Figure S3C). Furthermore, ectopically expressed FBXO16 degraded the nuclear pool of β‐catenin even in presence of Wnt3a stimulation (Figure 3D). Similar results were also obtained in the constitutively Wnt‐activated colon cancer cell line HCT116 (see supplementary material, Figure S3D).

A previous study showed that EGF also activates β‐catenin signaling 10. Interestingly, FBXO16 inhibited β‐catenin transactivation, even upon EGF stimulation (Figure 3E), which is supported by TOP/FOP assay (Figure 3F). Moreover, transcriptional activation by ectopically expressed β‐catenin was significantly reduced on the coexpression of FBXO16 (see supplementary material, Figure S3E). Thus, collectively, our results suggest that FBXO16‐mediated degradation of nuclear β‐catenin is not overridden by upstream Wnt/EGF signaling.

These data advocate that FBXO16 promotes the degradation of nuclear β‐catenin to ablate its transactivation activity. However, as a final confirmatory experiment to make our inference irrefutable, we carried out ChIP assays with β‐catenin after overexpressing FBXO16 in MDA‐MB‐231 cells. Results showed that FBXO16 abated the recruitment of β‐catenin on the promoter of its target genes, such as *MYC* and *CCND1* (see supplementary material, Figure S3F).

### FBXO16 maintains physiological levels of β‐catenin

**Figure 4:**
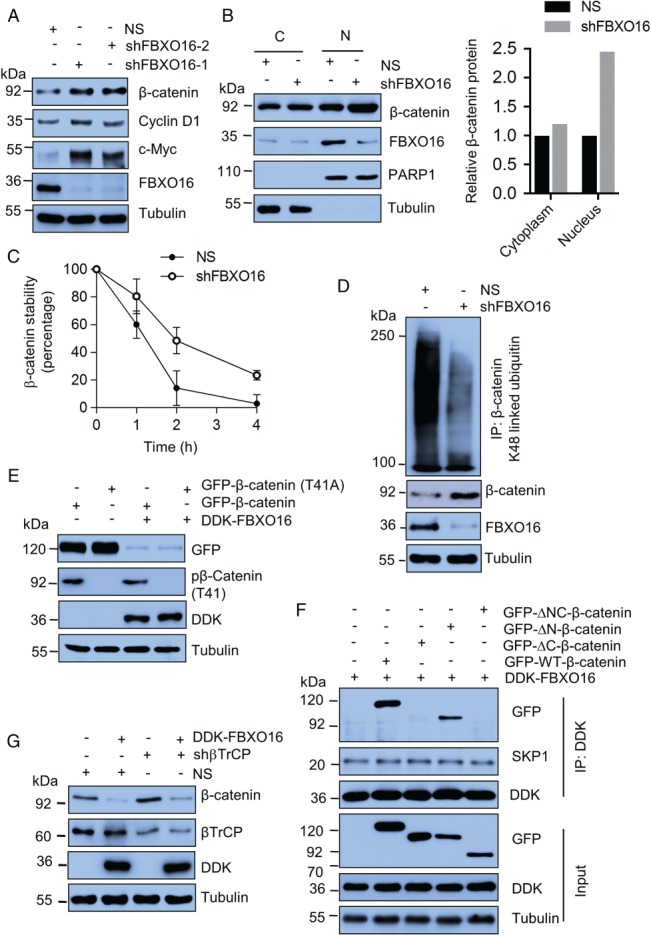
FBXO16 maintains the cellular level of β‐catenin independent of GSK3β and βTrCP. (A) Whole‐cell lysates of MCF7 cells expressing either NS or FBXO16 shRNAs were immunoblotted for the indicated proteins (*n* = 4). (B) Cytoplasmic (C) and nuclear (N) extracts of MCF7 cells stably expressing NS and *FBXO16* shRNA were immunoblotted for indicated proteins. PARP1 and tubulin were used as nuclear and cytoplasmic loading controls, respectively (*n* = 3). Right panel shows the quantification of relative levels of β‐catenin of left panel. Levels were normalized with respect to the loading control. (C) Levels of β‐catenin (in the cycloheximide assay, see supplementary material, Figure S4D) were quantified and normalized with loading control. Expression levels of β‐catenin were then normalized to 100% at time 0 h. (D) Whole‐cell protein extracts of MCF7 cells stably expressing either NS or *FBXO16* shRNA were immunoprecipitated using an anti‐β‐catenin antibody. Immunoprecipitates and input lysates were immunoblotted for the indicated proteins (*n* = 3). (E) Whole‐cell lysates of MCF7 cells, coexpressing either GFP‐β‐catenin and vector or GFP‐β‐catenin and DDK‐FBXO16 or GFP‐T41A‐β‐catenin and vector or GFP‐T41A‐β‐catenin and DDK‐FBXO16, were immunoblotted for the indicated proteins (*n* = 5). (F) Whole‐cell protein extracts of cells coexpressing the indicated proteins were immunoprecipitated with anti‐DDK antibody. Immunoprecipitates and input lysates were immunoblotted for the indicated proteins (*n* = 2). At 40 h posttransfection, cells were incubated with 5 μm MG132 for 6 h and harvested for cell lysate preparation. (G) MCF7 cells stably expressing either NS or βTrCP shRNA were transfected with either vector control or DDK‐FBXO16 for 48 h. Whole‐cell protein extracts were immunoblotted for the indicated proteins (*n* = 3).

Our findings suggested that ectopically expressed FBXO16 directs the proteasomal degradation of β‐catenin. Next, we questioned whether FBXO16 maintains the basal level of β‐catenin. Depletion of FBXO16 in MCF7 cells using two unrelated shRNAs resulted in increased β‐catenin levels (Figure 4A), with no apparent change at mRNA levels (see supplementary material, Figure S4A). Likewise, we observed similar findings in unrelated HEK‐293 cells (see supplementary material, Figure S4B). Furthermore, the expression of β‐catenin target genes *CCND1* and *MYC* was significantly elevated in FBXO16‐depleted cells (Figure 4A) at the transcript level (see supplementary material, Figure S4A). Consistent with these findings, the ChIP assay demonstrated that depletion of FBXO16 resulted in increased β‐catenin recruitment on the promoters of *CCND1* and *MYC* genes (see supplementary material, Figure S4C).

FBXO16 depletion selectively increased nuclear β‐catenin levels (Figure 4B). Furthermore, cycloheximide chase data showed that the half‐life of β‐catenin was increased in FBXO16 knockdown cells compared to the NS cells (Figure 4C, supplementary material, Figure S4D). To examine whether the depletion of FBXO16 attenuated the polyubiquitination of β‐catenin, we examined K48‐linked ubiquitin levels and found a drastic decrease in polyubiquitinated β‐catenin upon FBXO16 depletion (Figure 4D). Collectively, these results suggest that FBXO16 maintains the basal levels of nuclear β‐catenin at the proteasomal level.

### FBXO16‐mediated degradation of β‐catenin is independent of GSK3β, PKCδ, and βTrCP

Typically, F‐box proteins recognize phosphorylated substrates as a mark for promoting their ubiquitylation 32. It is paradigmatic that GSK3β‐imparted phosphorylation on threonine 41 of β‐catenin conduces its proteasomal degradation 33. So, we questioned whether FBXO16‐mediated β‐catenin degradation is GSK3β dependent. Results demonstrated that, despite administering GSK3β inhibitor BIO, FBXO16 degraded β‐catenin (see supplementary material, Figure S4E). To further substantiate this observation, we ectopically coexpressed the T41A mutant of GFP‐β‐catenin and FBXO16 in MCF7 and found that FBXO16 degraded the phosphorylation‐defective mutant of β‐catenin equivalently to wild type (Figure 4E), indicating that GSK3β‐mediated β‐catenin phosphorylation is not required for its recognition and degradation by FBXO16.

A recent study showed that PKCδ also plays an important role in β‐catenin degradation 19. Hence, we checked PKCδ's role in this aspect and found that FBXO16‐mediated β‐catenin degradation was independent of PKCδ (see supplementary material, Figure S4F). Moreover, we found that the interaction between β‐catenin and FBXO16 was phosphorylation‐independent (see supplementary material, Figure S4G).

We found that FBXO16 interacts physically with β‐catenin (Figure 1B). To identify the specific domain of β‐catenin that interacts with FBXO16, we used a series of β‐catenin deletion mutants (see supplementary material, Figure S4H). Results demonstrated that FBXO16 failed to degrade C‐terminal‐deleted β‐catenin (see supplementary material, Figure S4I). Next, we checked the interaction and found that FBXO16 does not interact with the C‐terminal deletion mutant (Figure 4F), indicating that the C‐terminal region of β‐catenin is indispensable for recognition by FBXO16.

βTrCP was the first discovered F‐box protein to degrade β‐catenin 17. Hence, we asked whether FBXO16‐mediated degradation of β‐catenin was βTrCP dependent. Immunoblotting data showed that ectopically expressed FBXO16 degraded β‐catenin equally in both NS‐ and βTrCP‐depleted MCF7 cells (Figure 4G). Collectively, our results demonstrated that FBXO16‐mediated β‐catenin degradation might be independent of GSK3β, PKCδ, and βTrCP.

### FBXO16 inhibits EMT by regulating β‐catenin levels

β‐Catenin is an exemplary regulator of EMT in development and diseases 34. Our results convincingly demonstrated that FBXO16 promotes degradation of nuclear β‐catenin and vetoes upstream controller Wnt signaling as well. This cellular interplay stoked us into assessing the expression of EMT hallmarks in the presence of FBXO16.

**Figure 5:**
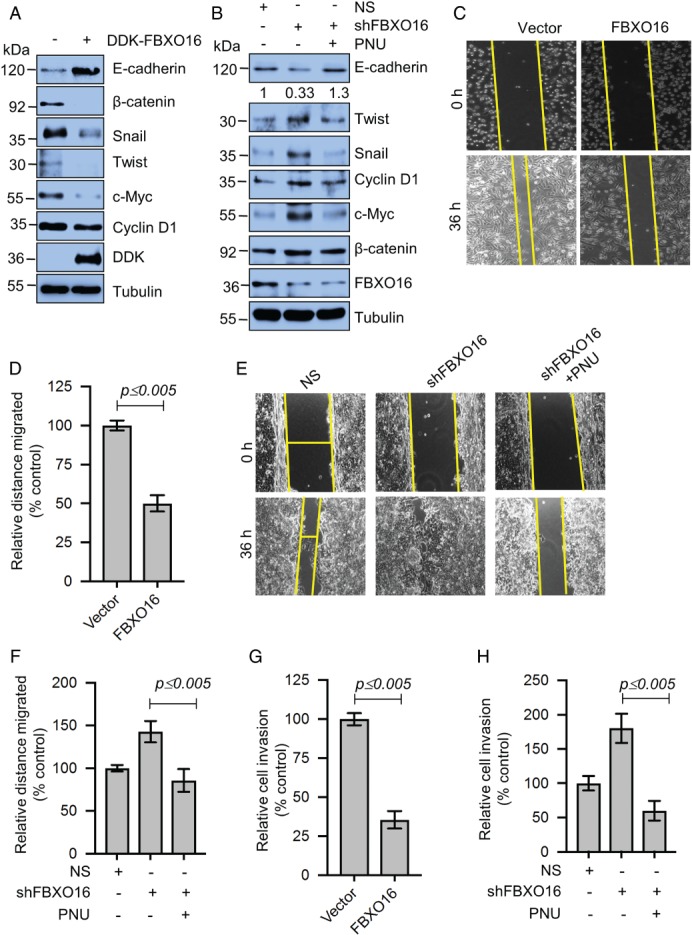
FBXO16 prevents EMT by limiting the expression of EMT regulators through proteasomal degradation of β‐catenin. (A) Whole‐cell lysates of MDA‐MB‐231 cells expressing either vector control or DDK‐FBXO16 were immunoblotted for the indicated proteins (*n* = 3). (B) Whole‐cell protein extracts of MCF7 cells stably expressing either NS or *FBXO16* shRNA were immunoblotted for the indicated proteins (*n* = 3). Cells were grown in the absence or presence of 500 nm PNU (β‐catenin inhibitor) for 36 h. (C) Scratch wound‐healing cell migration assay of MDA‐MB‐231 cells expressing either vector control or FBXO16. Cells were transfected for 48 h; then, wounds were created, and healing was tracked for 36 h (*n* = 3). (D) Quantitative data of scratch wound healing of three independent experiments. Wound healing with vector was normalized to 100%. Data are presented as mean ± SD. (E) Scratch would healing of MCF7 cells expressing either NS or *FBXO16* shRNA for 30 h in the absence or presence of 500 nm PNU (*n* = 3). (F) Quantitative data of scratch wound healing of three independent experiments. Wound healing of NS was normalized to 100%. Data are presented as mean ± SD. (G) Invasion of MDA‐MB‐231 cells expressing either vector control or DDK‐FBXO16 (*n* = 3). (H) Invasion of MCF7 cells expressing either NS or *FBXO16* shRNA in the absence or presence of 500 nm PNU (*n* = 3).

Ectopic expression of FBXO16 in MDA‐MB‐231 cells leads to the downregulation of mesenchymal markers like Snail and Twist, with concomitant upregulation of epithelial markers like E‐cadherin (Figure 5A). In contrast, silencing FBXO16 in MCF7 cells resulted in mesenchymal morphology (see supplementary material, Figure S5A) and increased the expression of EMT regulators (Figure 5B), which was restored using the β‐catenin‐TCF/LEF binding inhibitor, PNU. Another replica was performed using β‐catenin shRNA as a substitute of PNU (see supplementary material, Figure S5B) to consolidate the finding that FBXO16 prevents β‐catenin‐mediated EMT. As EMT is a key process of metastasis 35, we looked into migratory potential and invasiveness of the cells following overexpression/depletion of FBXO16. Ectopic expression of FBXO16 significantly suppressed scratch wound healing compared to the vector‐transfected cells (Figure 5C,D), and the converse was found for FBXO16‐depleted cells (Figure 5E,F). Furthermore, the addition of PNU suppressed the migratory potential of FBXO16 depleted cells, suggesting that elevated levels of β‐catenin in FBXO16‐depleted cells are responsible for enhanced cell migration (Figure 5E,F). Similar results were also observed following codepletion of FBXO16 and β‐catenin (see supplementary material, Figure S5C,D).

In addition, we examined the invasion of MDA‐MB‐231 cells following ectopic expression of FBXO16, and our results showed that ectopically expressed FBXO16 can potently suppress the invasion of MDA‐MB‐231 cells (Figure 5G). Conversely, depletion of FBXO16 in MCF7 cells led to significantly enhanced invasion, which was robustly inhibited following treatment with PNU (Figure 5H). Collectively, we found that FBXO16 restricts cell migration and invasion by limiting the expression of β‐catenin.

### FBXO16 inhibits tumorigenesis

**Figure 6:**
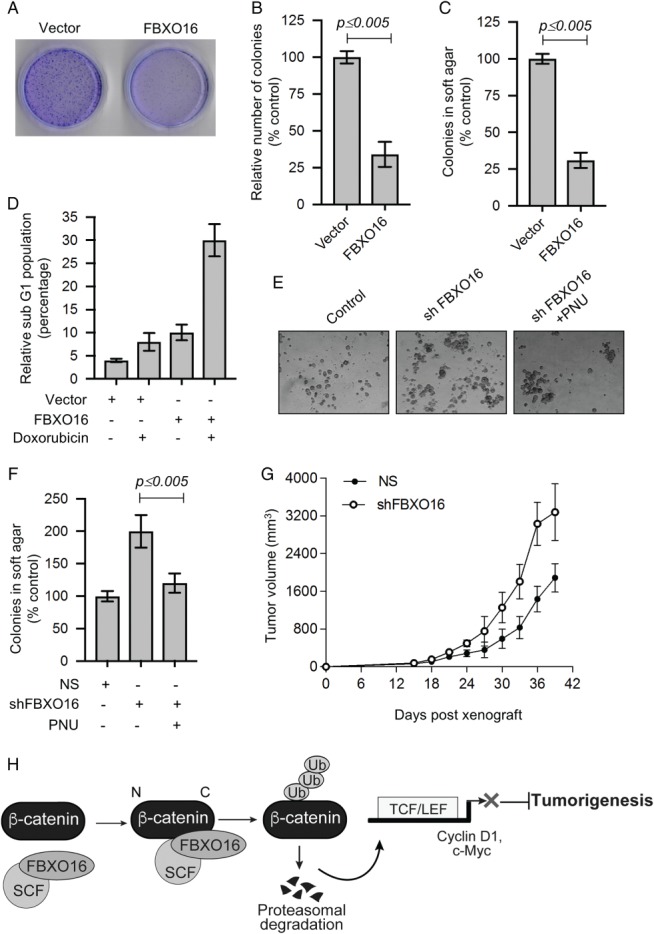
FBXO16 functions as putative tumor suppressor. (A) Long‐term colony formation of MDA‐MB‐231 cells expressing either vector control or DDK‐FBXO16. A total of 5000 transfected cells were seeded and allowed to grow for 15 days, and then, colonies were stained with crystal violet. The image represents one of the three biological replicates. (B) Number of colonies (from three independent experiments of panel A) were counted using ImageJ software and normalized to 100% for vector‐transfected cells. Data plotted as mean ± SD. (C) Soft agar colony formation assay of MDA‐MB‐231 cells expressing either vector control or DDK‐FBXO16. A total of 5000 transfected cells were seeded and allowed to grow for 21 days. Cells were then stained with crystal violet solution. Colonies were counted and normalized to 100% for vector control cells. Data are mean ± SD from three independent experiments. (D) Relative fraction of sub‐G0/G1 cell populations plotted with indicated treatments. (E) Soft agar colony formation assay of MCF7 cells expressing either NS or *FBXO16* shRNA in the presence or absence of the β‐catenin inhibitor 500 nm PNU. A total of 5000 cells were used for this assay (*n* = 3). (F) Number of colonies of soft agar assay (as in panel E) was counted and normalized to 100% for NS cells. Data are mean ± SD (*n* = 3). (G) NOD‐SCID mouse xenograft growth of MCF7 cells expressing either NS or *FBXO16* shRNA. Five mice were used for each group. (H) Model depicting the tumor‐suppressive activity of FBXO16 through the regulation of β‐catenin.

The nuclear accumulation of β‐catenin is a hallmark of malignant progression through the activation of many oncogenes 16, 19, 34. We found that the mRNA levels of crucial oncogenes, such as *CCND1* and *MYC,* were increased in FBXO16‐depleted cells because of increased β‐catenin levels (see supplementary material, Figure S4A). Hence, we performed a series of experiments to check whether FBXO16 has any role in cancer cell proliferation, either by its overexpression/depletion. Long‐term (Figure 6A,B) and soft agar (Figure 6C) colony formation assays demonstrated that numbers of colonies were significantly attenuated upon ectopic expression of FBXO16.

MDA‐MB‐231 cells are less sensitive to chemotherapeutic agents such as doxorubicin. We therefore examined whether FBXO16 has any effect on the sensitivity to chemotherapeutic drugs. Results demonstrated that the ectopic presence of FBXO16 leads to a significant increase in cell death upon doxorubicin treatment (Figure 6D).

Next, we examined cancer cell line growth following depletion of FBXO16 in MCF7 cells. Soft agar assays demonstrated that depletion of FBXO16 resulted in the formation of more colonies, which was notably inhibited by PNU (Figure 6E,F). A similar result was recapitulated following codepletion of β‐catenin in FBXO16 knockdown cells (see supplementary material, Figure S6A–C). These encouraging observations prompted us to further check tumor growth as xenografts in NOD‐SCID mice. Our results demonstrated that tumor growth was markedly increased following depletion of FBXO16 (Figure 6G, supplementary material, Figure S6D). Furthermore, IHC showed significantly increased levels of β‐catenin in FBXO16‐depleted mouse xenografts (see supplementary material, Figure S6E). Collectively, our findings suggested that FBXO16 may function as a putative tumor suppressor by limiting the activity of nuclear β‐catenin.

## Discussion

F‐box proteins form SCF complexes to target a wide range of cellular proteins for their polyubiquitination 20, 32. They are involved in cell cycle progression, DNA damage response, and cellular signaling. Therefore, deregulation of an F‐box protein is often associated with cancer. For instance, F‐box protein FBXW7 functions as a tumor suppressor by promoting proteasomal degradation of c‐Myc, Cyclin E, and Notch, whereas SKP2 functions as an oncogene by promoting degradation of tumor suppressors and activating AKT signaling by abrogating its proteasomal degradation 20. So, it is of prime importance to decipher the cellular functions of F‐box proteins for the development of novel targeted therapeutics.

*FBXO16* is located at human chromosome 8p12 36. However, the cellular function of FBXO16 is unknown. Here, for the first time, we have identified cellular targets of FBXO16 through mass spectrometry and unraveled its cellular function. We show that FBXO16 functions as a putative tumor suppressor by abrogating the function of nuclear β‐catenin (Figure 6H). It maintains the nuclear levels of β‐catenin. Depletion of FBXO16 resulted in increased malignancy because of aberrant nuclear accumulation of β‐catenin, emphasizing that elevated levels of FBXO16 in higher grades of breast cancer could be therapeutically beneficial to abrogate the β‐catenin driven malignancy. It is further supported by a database of breast cancer patient samples coupling overall survival of the patient with higher levels of FBXO16 (Figure 1F).

A recent study has found that aberrant nuclear accumulation of β‐catenin contributes to the malignancy in 50% of breast cancer cases 5. Aberrant nuclear accumulation of β‐catenin elevates the expression of numerous oncogenes associated with cellular proliferation. Likewise, it also promotes EMT by increasing the expression of transcription factors essential to metastasis. Therefore, attenuating nuclear accumulation of β‐catenin might be a novel strategy for restricting malignancy.

Recently, it has been suggested that TRIM33 directs proteasomal degradation of nuclear β‐catenin in a PKCδ‐dependent manner 19. However, nuclear localization of PKCδ is signal dependent 37. Moreover, we found that FBXO16 also facilitates proteasomal degradation of nuclear β‐catenin even in the absence of PKCδ, indicating that FBXO16‐mediated β‐catenin regulation could be different from that of TRIM33.

A previous study demonstrated that F‐box protein βTrCP promotes the degradation of β‐catenin in a GSK3β‐dependent manner. We found that FBXO16 facilitates the degradation of β‐catenin even in the absence of GSK3β signaling. Furthermore, βTrCP targets cytoplasmic pools of β‐catenin, whereas FBXO16 targets nuclear pools of β‐catenin. Moreover, βTrCP fails to degrade β‐catenin following activation of Wnt/EGF; in contrast, FBXO16‐mediated degradation of β‐catenin is independent of Wnt/EGF activation, indicating that FBXO16‐mediated regulation of β‐catenin might be different from βTrCP. Thus, FBXO16 is the first F‐box protein that facilitates degradation of nuclear β‐catenin, thereby inhibiting the cancer cell proliferation, migration, invasion *in vitro,* and tumor formation in NOD‐SCID mice.

A significant inverse relationship of FBXO16 and β‐catenin in breast cancer patient samples was observed. A recent report indicated a deletion of 8p21.1 in breast cancer, which is the chromosomal location of FBXO16 38. These findings indicate that downregulation of FBXO16 in breast cancer might unleash the oncogenic activity of β‐catenin. Thus, the activation of FBXO16 expression could be a novel therapeutic strategy in preventing cancers expressing the aberrant nuclear accumulation of β‐catenin.

## Author contributions statement

MKS and DP contributed to conception and design. MKS, DP, BM, SI, RKM, PKS, SR, and SKM contributed to writing of the manuscript. MKS, DP, SI, and RKM contributed to the development of methodology. DP, SI, RKM, and USD contributed to the acquisition of data. MKS, DP, RKM, SKM, RB, and SR contributed to the interpretation of data (e.g. statistical analysis, biostatistics, computational analysis).

**SUPPLEMENTARY MATERIAL ONLINE**

**Supplementary materials and methods**

**Supplementary figure legends**

**Figure S1.** Analysis of immunoprecipitates

**Figure S2.** FBXO16 regulates β‐catenin

**Figure S3.** FBXO16 maintains basal levels of β‐catenin

**Figure S4.** FBXO16 regulates β‐catenin in phosphorylation independent manner

**Figure S5**. Depletion of FBXO16 promotes EMT

**Figure S6**. Depletion of FBXO16 promotes tumorigenesis.

**Table S1.** Primers used for generating F‐box deleted FBXO16

**Table S2.** Primers used for RT‐qPCR

**Table S3.** Primers used for ChIP assays

**Table S4.** Interactomes of FBXO16 (minimum three unique peptides were identified for each protein)

## Supporting information


**Supplementary materials and methods**

**Supplementary figure legends**

**Figure S1.** Analysis of immunoprecipitates

**Figure S2.** FBXO16 regulates β‐catenin

**Figure S3.** FBXO16 maintains basal levels of β‐catenin

**Figure S4.** FBXO16 regulates β‐catenin in phosphorylation independent manner

**Figure S5.** Depletion of FBXO16 promotes EMT

**Figure S6.** Depletion of FBXO16 promotes tumorigenesis

**Table S1.** Primers used for generating F‐box‐deleted FBXO16
**Table S2.** Primers used for RT‐qPCR
**Table S3.** Primers used for ChIP assays
**Table S4.** Interactomes of FBXO16 (a minimum of three unique peptides were identified for each protein)
