# Supplementary material for: F‐box protein FBXO16 functions as a tumor suppressor by attenuating nuclear β‐catenin function
Source: J Pathol. 2019 Mar 8;248(3):266–79. doi: 10.1002/path.5252 (PMC6619347; doi:10.1002/path.5252)
Supplement: Supplementary file 1 — Supplementary materials and methods [file PATH-248-266-s008.docx]

**F-box protein FBXO16 functions as a tumor suppressor by attenuating nuclear β-catenin function**

Paul D *et al*. *J Pathol* DOI: 10.1002/path.5252

**Supplementary materials and methods**

Reference numbers refer to the main text list

**Identification of FBXO16 interactomes by mass spectrometry**

MCF7 cells expressing either vector control or DDK-FBXO16 were incubated with 5 μM of MG132 for 6 h prior to harvesting. Cells were lysed in buffer (50 mM Tris-HCL pH 7.4, 200 mM NaCl, 50 mM NaF, 1 mM Na_3_VO_4_, 0.5% Triton X-100 and protease inhibitor cocktail) on ice for 30 min [23] and seven independent immunoprecipitation reactions each for vector and FBXO16 were processed as described for Co-immunoprecipitation. The immunoprecipitates were eluted from the beads using elution buffer (8 M urea and 0.5 M Tris, pH 7.4) for 10–15 min and collected. The eluted samples were then precipitated using acetone. Cold acetone (HPLC grade, Sigma, St Louis, MO, USA) was added to the protein sample in 4:1 ratio and the mixture was vortexed. The mixture was then incubated overnight at -20 ºC and then the precipitate was centrifuged at 4 ºC for 20 min at 12000× *g*. The pellet obtained was air dried and immediately processed for in-solution digestion. The pellet was resuspended in 100 mM triethyl ammonium bicarbonate (Sigma) and trypsin digestion was carried out by using sequencing grade trypsin (Promega, USA) by adding 1: 20 ratio of trypsin and protein. The sample was incubated at 37 ºC water bath for 16 h. Digestion was then stopped and peptide solution was dried and dissolved in 20 μl water containing 0.1% formic acid. LC- MS/MS analyses of peptide mixture was performed using Orbitrap fusion mass spectrometer (ThermoFisher Scientific, Waltham, MA, USA).

***In vitro* ubiquitination assay**

For *in vitro* ubiquitination assay, recombinant GST- β-catenin was used incubated with 0.25 μM UBE1, 0.25 μM UBCH3, 0.25 μM UBCH5C, 0.1 μM ubiquitin aldehyde, 2.5 μg/μL ubiquitin (all from Boston Biochem) in a reaction buffer containing (25 mM Tris-HCl pH7.5, 1 mM KCl, 1 mM DTT 2.5 mM MgCl_2_ 2.5 mM ATP) in presence or absence of immunopurified SCF^FBXO16.^ The ubiquitylation reaction was carried at 30 °C for 2 h, and the assay mixture was resolved on SDS page followed by immunoblotting with anti GST antibody (Cell Signaling).
