# Supplementary material for: F‐box protein FBXO16 functions as a tumor suppressor by attenuating nuclear β‐catenin function
Source: J Pathol. 2019 Mar 8;248(3):266–79. doi: 10.1002/path.5252 (PMC6619347; doi:10.1002/path.5252)
Supplement: Supplementary file 2 — Supplementary figure legends [file PATH-248-266-s009.docx]

**F-box protein FBXO16 functions as a tumor suppressor by attenuating nuclear β-catenin function**

Paul D *et al*. *J Pathol* DOI: 10.1002/path.5252

**Supplementary figure legends**

**Figure S1. Analysis of immunoprecipitates.** (A) Mass spectrometric analysis of FBXO16 immunoprecipitates showing unique peptide sequences of β-catenin. (B) Whole cell lysates of HEK-293 cells were immunoprecipitated either with anti-β-catenin antibody or IgG. Immunoprecipitates and input lysates were separated on SDS-PAGE and immunoblotted for the indicated proteins (n=2). Cells were grown in the absence and presence of 5 µM MG132 for 6 h before harvesting. (C) *In vitro* interaction of FBXO16 and β-catenin. Purified GST-β-catenin was incubated in the absence or presence of His-FBXO16 for 30 min at 4 °C, then the protein mixtures were immunoprecipitated with ant-GST antibody. Immunoprecipitates and input protein mixture were immunoblotted with indicated antibody. (D) Co-localization of DDK-FBXO16 and DDK-ΔNLS-FBXO16 with GFP-β-Catenin in MCF7 cells by immunofluorescence study. (E) Expression levels of β-catenin (bottom row) and FBXO16 (middle row) in different ER/PR/HER2 sub-types of breast cancer patient samples. Tissues were stained for immunohistochemical analysis as described in Materials and Methods. (F) Relative DNA copy number of FBXO16 in different breast cancer cell lines (Source – Oncomine data base). (G) Kaplan–Meier plot depicting distance free metastatic survival of breast cancer patient cohorts of TNBC, ER^-^PR^+^HER2^+^, ER^-^PR^-^HER2^+^ sub-types. Red trace represents comparative higher expression of FBXO16.

**Figure S2.** **FBXO16 regulates β-catenin.** (A) Graph represents the quantification of relative levels of β-catenin of Figure 2B. Levels were normalized with respect to the loading control. (B) MDA-MB-231 cells expressing either vector control or DDK-FBXO16 were analyzed for mRNA levels of *CTNNB1* by RT-qPCR. The results (mean ± SD of 3 independent experiments) are expressed as relative mRNA levels of β-catenin. mRNA levels of β-catenin were normalized with the mRNA levels of *ACTB*. “ns” represents statistically not significant. (C) MDA-MB-231 cells expressing either vector control or DDK-FBXO16 for 36 h were then exposed to 40 μg/mL Cycloheximide (CHX) for indicated time periods. Whole cell protein extracts were immunoblotted for indicated proteins (n=3). (D) MDA-MB-231 cells were transfected either with vector or FBXO16 for 36 h. Transfected cells were then treated with 5 μM MG132 for 6 h prior to harvesting. Whole cell lysates were immunoprecipitated with an anti-β-catenin antibody. Immunoprecipitates and input protein extracts were revolved in SDS-PAGE and blotted for indicated proteins (n=3).

**Figure S3. FBXO16 maintains basal levels of β-catenin.** (A) MDA-MB-231 cells expressing either vector control or DDK-FBXO16 or DDK-∆F-FBXO16 for 36 h. Transfected cells were then incubated with 5 μM MG132 for 6 h before harvesting. Whole cell protein extracts were immunoprecipitated with anti-β-catenin antibody. Immunoprecipitates and input protein extracts were immunoblotted for indicated proteins (n=3). (B) Cytoplasmic and nuclear fractions of cells ectopically expressing either DDK-FBXO16 or DDK-ΔNLS-FBXO16 were immunoblotted for indicated proteins. (C) Cells ectopically expressing either vector or DDK-FBXO16 were stimulated with Wnt3a for 12 h. Whole cell extracts were immunoblotted for indicated proteins. (D) Whole cell protein extracts of HCT116 cells expressing either vector control or DDK-FBXO16 for 48 h were immunoblotted for the indicated proteins. (E) MDA-MB-231 cells were transfected with the indicated plasmids including TOP/FOP and pRL-TK. Transfection efficiency was normalized using the pRL-TK reporter. Luciferase activity was measured at 48 h post transfection. Luciferase activity is shown as a ratio of TOP and FOP (n=3). (F) ChIP assay performed to examine the recruitment of β-catenin on the promoter of *CCND1* and *c-Myc* following overexpression of FBXO16 (n=3).

**Figure S4**. **FBXO16 regulates β-catenin in phosphorylation independent manner.** (A) Relative mRNA levels of *MYC, CCND1* and *CTNNB1* in MCF7 cells expressing either NS or *FBXO16* shRNA. Cells were incubated with and without 500 nM PNU for 24 h. Total RNA was isolated, cDNA was prepared and qPCR was performed using SYBR green (n=3). (B) Whole cell lysates of HEK-293 cells expressing either NS or *FBXO16* shRNA were immunoblotted for indicated proteins (n=3). (C) ChIP assay was performed to assess the recruitment of β-catenin onto the promoter of *CCND1* and *MYC* in MCF-7 cells expressing NS or *FBXO16* shRNA (n=3). (D) MCF7 cells stably expressing either NS or *FBXO16* shRNA were treated with 40 μg/ml cycloheximide for indicated time periods. Whole cell protein extracts were immunoblotted for the indicated proteins (n=3). (E) Cells were transfected with either vector or DDK-FBXO16 for 36 h. Transfected cells were then grown in the presence of 125 nM BIO for 12 h, as indicated and whole cell lysates were then immunoblotted for the indicated proteins (n=3). (F) Whole cell protein extracts of MDA-MB-231 cells expressing either vector control or DDK-FBXO16 in the presence or absence of 50 nM PKCδ inhibitor were immunoblotted for indicated proteins (n=3). (G) Whole cell lysates of MCF7 cells expressing DDK-FBXO16 were either untreated or treated with lambda protein phosphatase for 30 min at 30 ºC. Cell lysates were then immunoprecipitated with anti-DDK antibody and immunoprecipitates were immunoblotted for the indicated proteins (n=3). (H) Schematic illustration of β-catenin domains and deletion constructs. (I) WT and deletion mutants of β-catenin were co-expressed either with vector or DDK-FBXO16 for 48 h. Whole cell protein extracts were immunoblotted for the indicted proteins (n=3).

**Figure S5. Depletion of FBXO16 promotes EMT.** (A) Pro-mesenchymal morphological changes (marked by arrows) in FBXO16 stable knockdown cells. Growing cells were imaged and representative images are shown. (B) Whole cell lysates of MCF7 cells stably expressing either NS or *FBXO16* or *FBXO16* and *CTNNB1* shRNAs were immunoblotted for the indicated proteins (n=3). (C) Scratch wound healing of MCF7 cells stably expressing either NS or *FBXO16* or *FBXO16* and *CTNNB1* shRNA (n=3). (D) Quantification of the distance migrated by cells in the scratch wound healing assay (depicted in B). Distance migrated by NS cells was normalized to 100%. Data is presented as mean ± SD of three independent experiments.

**Figure S6**. **Depletion of FBXO16 promotes tumorigenesis.** (A) Long term colony formation of MCF7 cells stably expressing either NS or *FBXO16* or *FBXO16* and *CTNNB1* shRNA. Five thousand cells were seeded and grown for 15 days and then stained with crystal violet solution (n=3). (B) Soft-agar colony formation assay of NS or *FBXO16* knockdown or *FBXO16* and *CTNNB1* double knockdown cells. Ten thousand cells were seeded and grown for 15 d and stained with crystal violet solution (n=3). (C) Quantification of number of colonies formed in soft agar colony formation assay (depicted in B). Number of colonies of NS cells were normalized to 100 percent. (D) NS and *FBXO16* knockdown MCF7 cells (5x10^6^) were injected subcutaneously in the flank of the NOD-SCID mice. When a palpable tumor formed, the tumor volume was measured on every third day until mice were sacrificed. (E) Expression levels of β-catenin and FBXO16 in mouse xenografts. Tissues were stained for immunohistochemical analysis as described in the Materials and Methods.
