# Supplementary figures and images for: F‐box protein FBXO16 functions as a tumor suppressor by attenuating nuclear β‐catenin function
Source: J Pathol. 2019 Mar 8;248(3):266–79. doi: 10.1002/path.5252 (PMC6619347; doi:10.1002/path.5252)

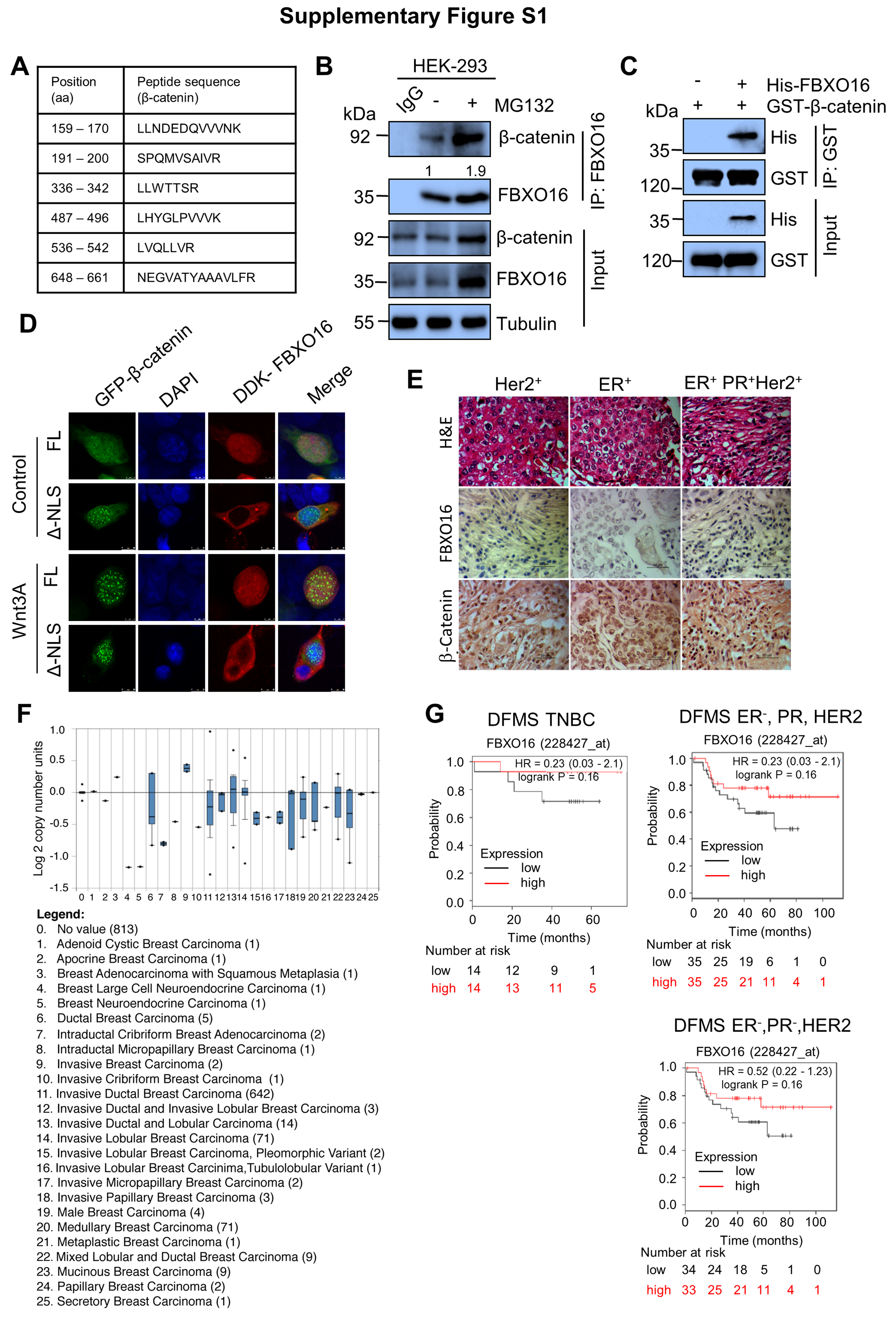

Supplement: Supplementary file 3 — Figure S1. Analysis of immunoprecipitates [file PATH-248-266-s001.tif]

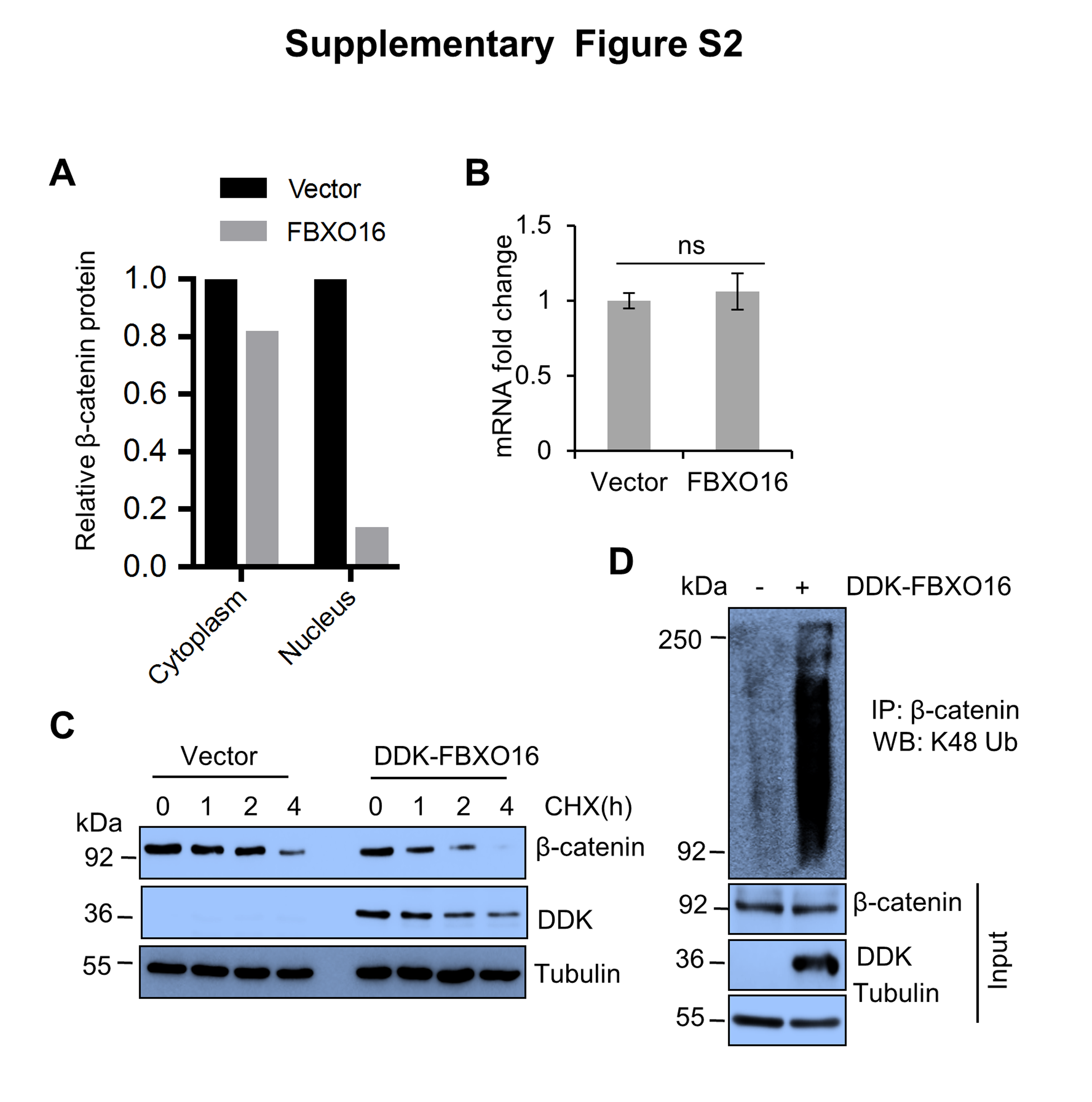

Supplement: Supplementary file 4 — Figure S2. FBXO16 regulates β‐catenin [file PATH-248-266-s002.tif]

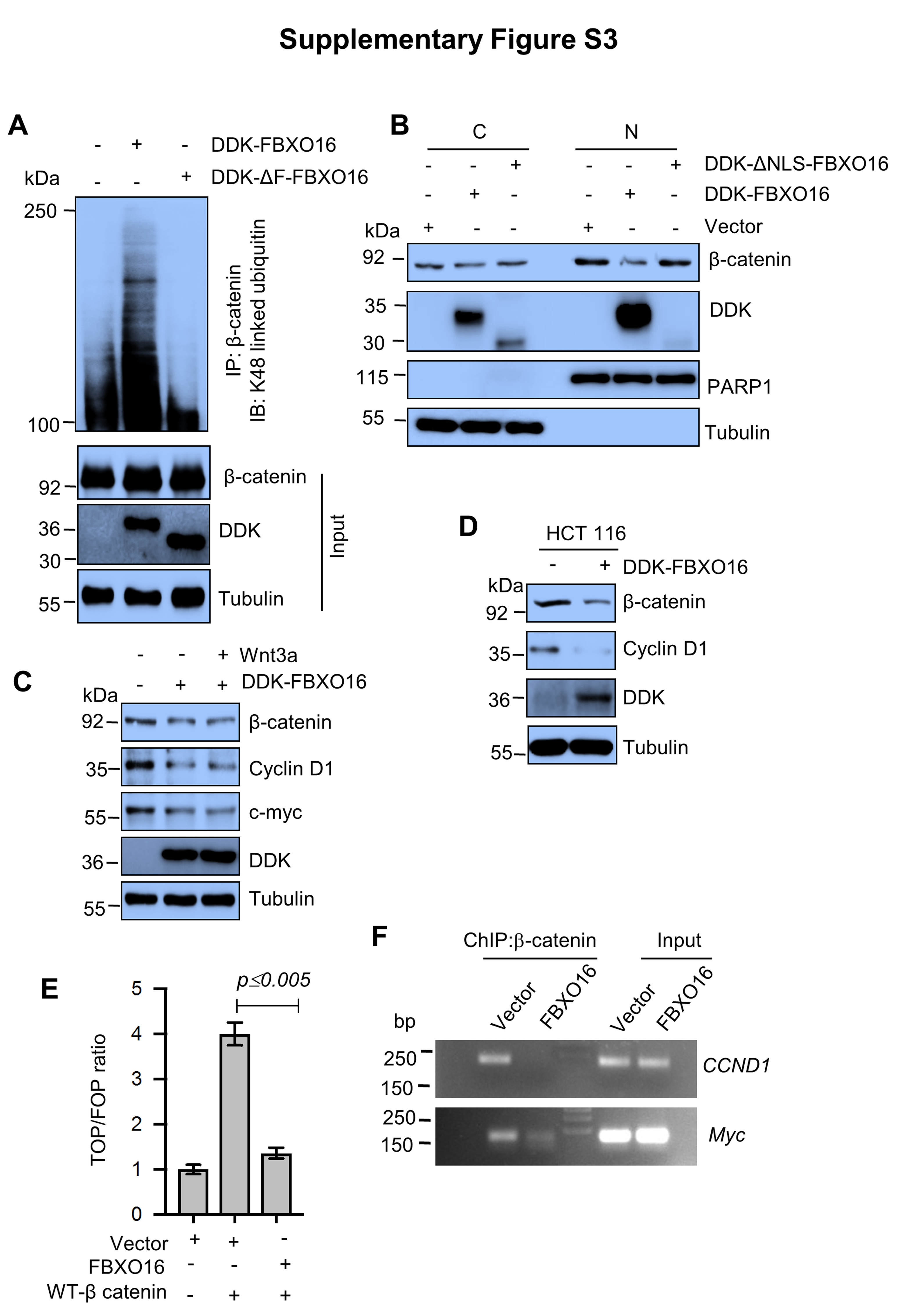

Supplement: Supplementary file 5 — Figure S3. FBXO16 maintains basal levels of β‐catenin [file PATH-248-266-s003.tif]

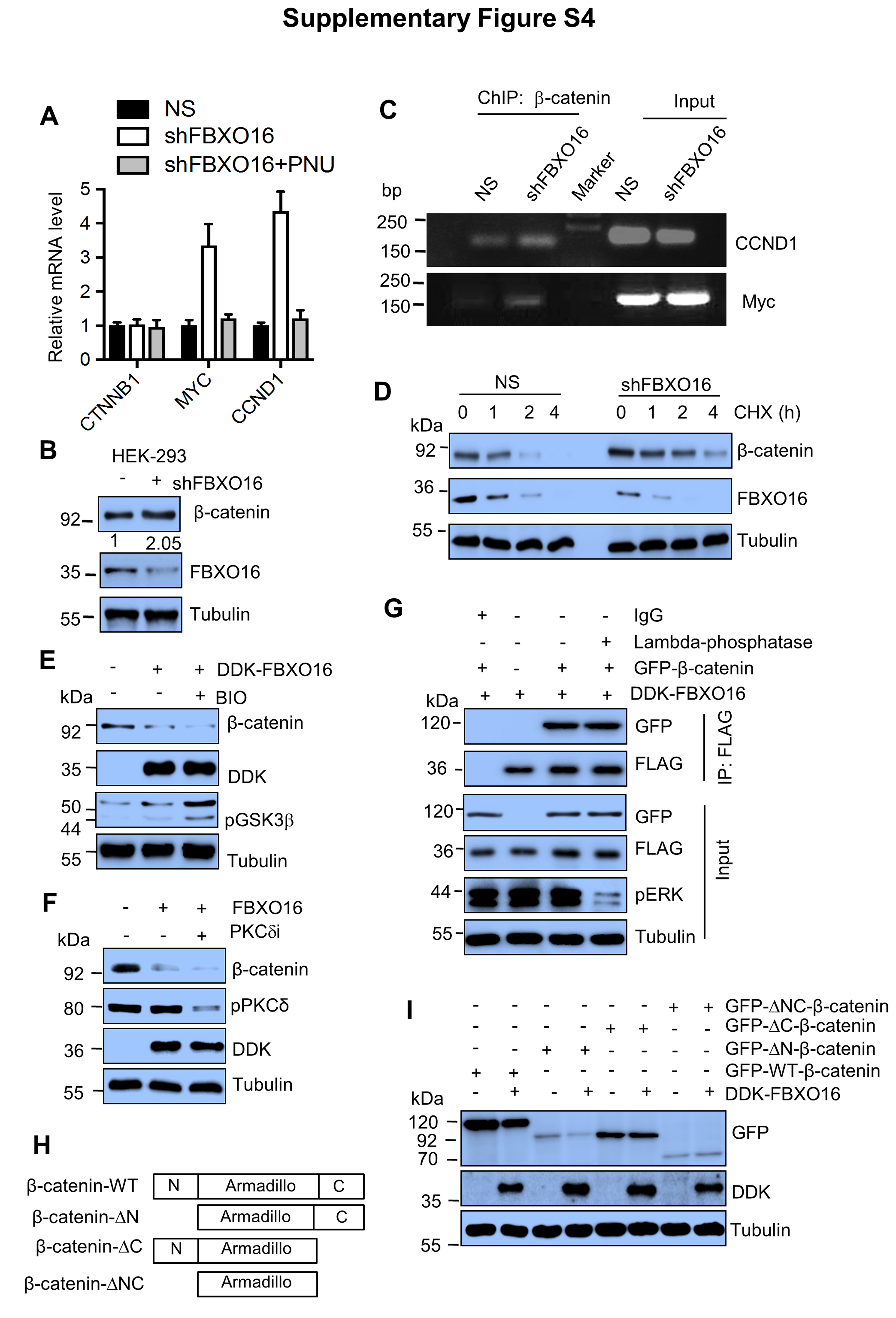

Supplement: Supplementary file 6 — Figure S4. FBXO16 regulates β‐catenin in phosphorylation independent manner [file PATH-248-266-s004.tif]

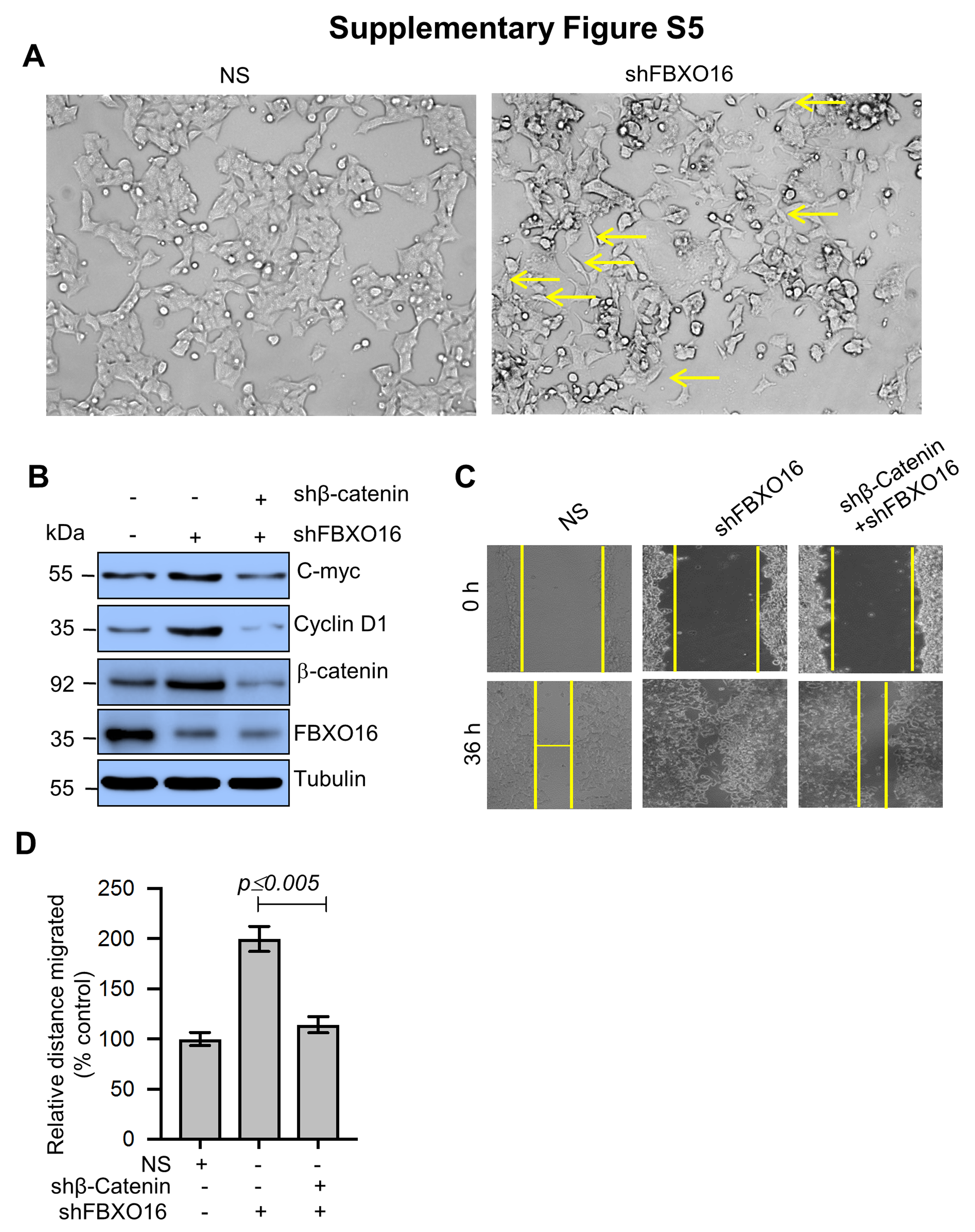

Supplement: Supplementary file 7 — Figure S5. Depletion of FBXO16 promotes EMT [file PATH-248-266-s005.tif]

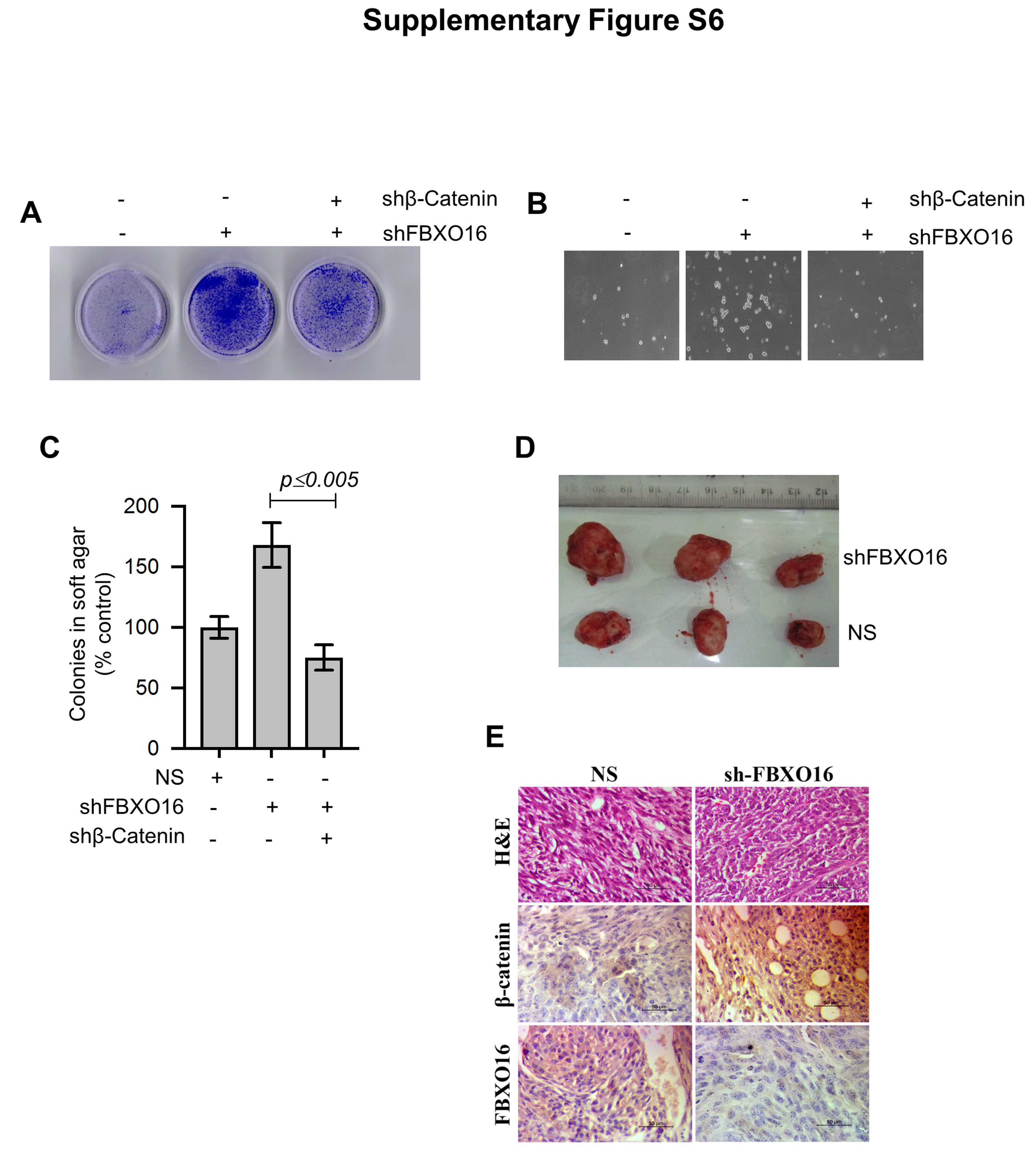

Supplement: Supplementary file 8 — Figure S6. Depletion of FBXO16 promotes tumorigenesis [file PATH-248-266-s006.tif]
