# Supplementary material for: F‐box protein FBXO16 functions as a tumor suppressor by attenuating nuclear β‐catenin function
Source: J Pathol. 2019 Mar 8;248(3):266–79. doi: 10.1002/path.5252 (PMC6619347; doi:10.1002/path.5252)
Supplement: Supplementary file 9 — Table S1. Primers used for generating F‐box‐deleted FBXO16 Table S2. Primers used for RT‐qPCR Table S3. Primers used for ChIP assays Table S4. Interactomes of FBXO16 (a minimum of three unique peptides were identified for each protein) [file PATH-248-266-s007.docx]

**F-box protein FBXO16 functions as a tumor suppressor by attenuating nuclear β-catenin function**

Paul D *et al*. *J Pathol* DOI: 10.1002/path.5252

**Supplementary Tables S1-S4.**

**Table S1. Primers used for generating F-box deleted FBXO16**

| Primer Name | Sequence (5’-3’) |
| --- | --- |
| ∆F-FBXO16 F | 5’ GCAGAAGCCTGTTTACGGTTTAAC 3’ |
| ∆F-FBXO16 R | 5’ CCGTAAACAGGCTTCTGCTGGAATTTTCTC 3’ |

**Table S2. Primers used for RT-qPCR**

| Primer Name | Sequence (5’-3’) |
| --- | --- |
| β-catenin F | GGTACCGAATCAAGCAGAGC |
| β-catenin R | GCAGTAGGAGGACGAGTTGG |
| Cyclin D1 F | TCTACACCGACAACTCCATCC |
| Cyclin D1 R | TTCCACTTGAGCTTGTTCACC |
| c-Myc F | AGCGACTCTGAGGAGGAACA |
| c-Myc R | CTCTGACCTTTTGCCAGGAG |
| GAPDH F | AATCCCATCACCATCTTCCA |
| GAPDH R | TGGACTCCACGACGTACTCA |

**Table S3. Primers used for ChIP assays**

| Primer Name | Sequence (5’-3’) |
| --- | --- |
| Cyclin D1 F | CACCTCCACCTCACCCCCTAAATCC |
| Cyclin D1 R | ACTCCCCTGTAGT CCGTGTGACGTT |
| c-Myc F | TTGCTGGGTTATTTTAATCAT |
| c-Myc R | ACTGTTTGACAAACCGCATCC |

**Table S4. Interactomes of FBXO16 (minimum three unique peptides were identified for each protein)**

| Sr. No | Protein Name | Gene symbol |
| --- | --- | --- |
| 1 | **F-box only protein 16** | **FBXO16** |
| 2 | Apical endosomal glycoprotein | MAMDC4 |
| 3 | Ataxin-2 | ATXN2 |
| 4 | Beta-1-syntrophin | SNTB1 |
| 5 | Cingulin-like protein 1 | CGNL1 |
| 6 | Ecto-NOX disulfide-thiol exchanger 2 | ENOX2 |
| 7 | Enhancer of mRNA-decapping protein 3 | EDC3 |
| 8 | F-box only protein 38 | FBXO38 |
| 9 | Hypoxia up-regulated protein 1 | HYOU1 |
| 10 | Inner nuclear membrane protein Man1 | LEMD3 |
| 11 | Insulin receptor substrate 1 | IRS1 |
| 12 | Isoform 2 of Uncharacterized protein C1orf167 | C1orf167 |
| 13 | Isoform 3 of DCC-interacting protein 13-beta | APPL2 |
| 14 | Isoform 4 of Double-stranded RNA-specific adenosine deaminase | ADAR |
| 15 | Isoform 4 of Latent-transforming growth factor beta-binding protein 1 | LTBP1 |
| 16 | Keratin, type II cuticular Hb2 | KRT82 |
| 17 | LIM and calponin homology domains-containing protein 1 | LIMCH1 |
| 18 | Neuron navigator 3 | NAV3 |
| 19 | Ninein-like protein | NINL |
| 20 | Partner and localizer of BRCA2 | PALB2 |
| 21 | Retinal guanylyl cyclase 1 | GUCY2D |
| 22 | Serine/threonine-protein kinase 24 (Fragment) | STK24 |
| 23 | Spectrin beta chain, non-erythrocytic 4 | SPTBN4 |
| 24 | Talin-1 | TLN1 |
| 25 | 1-phosphatidylinositol 3-phosphate 5-kinase | PIKFYVE |
| 26 | 26S protease regulatory subunit 7 | PSMC2 |
| 27 | 40S ribosomal protein S8 | RPS8 |
| 28 | Actin-related protein 2/3 complex subunit 3 | ARPC3 |
| 29 | Actin-related protein 3 | ACTR3 |
| 30 | Alpha-protein kinase 3 | ALPK3 |
| 31 | Ankyrin repeat domain-containing protein 17 | ANKRD17 |
| 32 | ATP-dependent RNA helicase DHX36 | DHX36 |
| 33 | BAG family molecular chaperone regulator 2 | BAG2 |
| 34 | BEN domain-containing protein 3 | BEND3 |
| 35 | CAD protein | CAD |
| 36 | Cadherin EGF LAG seven-pass G-type receptor 1 | CELSR1 |
| 37 | CAP-Gly domain-containing linker protein 3 | CLIP3 |
| 38 | **Catenin beta-1** | **CTNNB1** |
| 39 | Chromodomain-helicase-DNA-binding protein 7 | CHD7 |
| 40 | Clathrin heavy chain | CLTC |
| 41 | Collagen alpha-1(II) chain | COL2A1 |
| 42 | Collagen alpha-5(VI) chain | COL6A5 |
| 43 | Cytospin-A | SPECC1L |
| 44 | Death-inducer obliterator 1 | DIDO1 |
| 45 | DNA replication licensing factor MCM5 | MCM5 |
| 46 | DNA-dependent protein kinase catalytic subunit | PRKDC |
| 47 | DnaJ homolog subfamily B member 2 | DNAJB2 |
| 48 | Dystrophin | DMD |
| 49 | Eukaryotic initiation factor 4A-I | EIF4A1 |
| 50 | Fatty acid synthase | FASN |
| 51 | Fragile X mental retardation syndrome-related protein 1 | FXR1 |
| 52 | Gem-associated protein 4 | GEMIN4 |
| 53 | Glycine dehydrogenase (decarboxylating), mitochondrial | GLDC |
| 54 | GTP-binding nuclear protein Ran (Fragment) | RAN |
| 55 | Histone-binding protein RBBP4 | RBBP4 |
| 56 | Host cell factor 1 | HCFC1 |
| 57 | Inactive ubiquitin carboxyl-terminal hydrolase 54 | USP54 |
| 58 | Integrin alpha-7 | ITGA7 |
| 59 | Isoform 2 of Melanoma-associated antigen D1 | MAGED1 |
| 60 | Isoform 2 of Myosin-11 | MYH11 |
| 61 | Isoform 2 of Poly(rC)-binding protein 2 | PCBP2 |
| 62 | Isoform 2 of Protein NEDD1 | NEDD1 |
| 63 | Isoform 2 of Stress-induced-phosphoprotein 1 | STIP1 |
| 64 | Isoform 2 of Transketolase | TKT |
| 65 | Isoform 3 of Histone-lysine N-methyltransferase 2D | KMT2D |
| 66 | Isoform 3 of NUT family member 1 | NUTM1 |
| 67 | Isoform 3 of SPATS2-like protein | SPATS2L |
| 68 | Isoform 3 of Unconventional myosin-Va | MYO5A |
| 69 | Isoform 4 of M-phase inducer phosphatase 2 | CDC25B |
| 70 | Isoform 4 of YLP motif-containing protein 1 | YLPM1 |
| 71 | Isoform 8 of Erbin | ERBIN |
| 72 | Laminin subunit beta-2 | LAMB2 |
| 73 | Leucine-rich repeat-containing protein 7 | LRRC7 |
| 74 | LIM domain only protein 7 | LMO7 |
| 75 | Malate dehydrogenase, mitochondrial | MDH2 |
| 76 | Mediator of RNA polymerase II transcription subunit 12-like protein | MED12L |
| 77 | mRNA-decapping enzyme 1A | DCP1A |
| 78 | NCK-interacting protein with SH3 domain | NCKIPSD |
| 79 | Nesprin-1 | SYNE1 |
| 80 | Neuroblast differentiation-associated protein AHNAK | AHNAK |
| 81 | Neurofilament heavy polypeptide | NEFH |
| 82 | Oxygen-regulated protein 1 | RP1 |
| 83 | Pecanex-like protein 3 | PCNX3 |
| 84 | Pleckstrin homology domain-containing family H member 1 | PLEKHH1 |
| 85 | Potassium voltage-gated channel subfamily A member 10 | KCNA10 |
| 86 | Pre-mRNA-processing-splicing factor | PRPF8 |
| 87 | Probable ATP-dependent RNA helicase DDX46 | DDX46 |
| 88 | Probable E3 ubiquitin-protein ligase MARCH10 | Mar-10 |
| 89 | Prolactin-inducible protein | PIP |
| 90 | Proline-rich basic protein 1 | PROB1 |
| 91 | Protein FAM13A | FAM13A |
| 92 | Protein FAM193A | FAM193A |
| 93 | Protein FAM98C (Fragment) | FAM98C |
| 94 | Protein NYNRIN | NYNRIN |
| 95 | Protein O-GlcNAcase | MGEA5 |
| 96 | Protein RCC2 | RCC2 |
| 97 | Putative helicase MOV-10 | MOV10 |
| 98 | Regulator of nonsense transcripts 3B | UPF3B |
| 99 | RNA binding motif protein 10, isoform CRA_d | RBM10 |
| 100 | RuvB-like 2 | RUVBL2 |
| 101 | Serine/arginine-rich splicing factor 5 | SRSF5 |
| 102 | Serine/threonine-protein phosphatase PP1-beta catalytic subunit | PPP1CB |
| 103 | Signal peptide peptidase-like 2C | SPPL2C |
| 104 | Signal-induced proliferation-associated 1-like protein 1 | SIPA1L1 |
| 105 | Src substrate cortactin | CTTN |
| 106 | Suprabasin | SBSN |
| 107 | Synemin | SYNM |
| 108 | Tandem C2 domains nuclear protein | TC2N |
| 109 | Testis-expressed sequence 13C protein | TEX13C |
| 110 | Tetratricopeptide repeat protein 28 | TTC28 |
| 111 | Tropomodulin-2 | TMOD2 |
| 112 | Tubulin beta-4B chain | TUBB4B |
| 113 | Uncharacterized protein KIAA1211 | KIAA1211 |
| 114 | Unconventional myosin-IXb | MYO9B |
| 115 | Unconventional myosin-XVIIIa | MYO18A |
| 116 | Vimentin | VIM |
| 117 | WASH complex subunit 7 | KIAA1033 |
